# Regulations for Medical Graduation in the University of Dublin

**Published:** 1846-12

**Authors:** 


*Regulations for Medical graduation in the University of Dublin.*—The times of graduation are Shrove Tuesday and the first Tuesday in July. The medical examinations terminate the Tuesday of the preceding week. Candidates must previously have completed their medical education, and produced a chart testifying to the details of the same and subscribed by the registrar to the professors in the School of Physic, as well as by the persons signing the certificate.

1st. Candidates *who have graduated in arts* may obtain the degree of Bachelor of Medicine at any of the ensuing half-yearly periods of graduation, provided the requisite medical education and examinations shall have been accomplished. The payment at entrance is £15. The fees for study in arts, during four years, are £7 10s. each half year; and the fees for graduation in arts £8 17s. 6d.

The graduation fees for the degree of Bachelor of Medicine are £11 15s. The testimonium of the M. B. degree will contain the following certificate: "Testamur sedulam operam medicinæ navassæ et examinationes coram professoribus feliciter sustinuisse."

The medical education of a Bachelor of Medicine comprises attendance on the following courses of lectures, (of which three, at the discretion of the candidate, may be attended at the University of Edinburgh,) in the School of Physic established by act of Parliament, provided that one and not more than three of the courses which begin in November be attended during each of four sessions. The courses are—on anatomy and surgery, chemistry, botany, materia medica and pharmacy, institutes of medicine, practice of medicine, midwifery, (by the professor to the College of Physicians,) clinical lectures at Sir Patrick Dun's Hospital during at least one session of six months, as delivered by the professors in the School of Physic.

The fees for attendance on the clinical lectures are regulated by act of parliament—they amount to £3 3s. to the professors for each three months' attendance, and (provided the student be of two years' standing in the university) £3 3s. to the treasurer of the hospital for the first year, with a proportionate sum for any longer period. The fees for each of the other courses are £4 4s.

The examinations for the degree of Bachelor of Medicine are conducted by the Regius Professor of the University, the six professors of the School of Physic, and the Professor of Midwifery to the College of Physicians.

No further examination is requisite for the degree of Doctor of Medicine, it may be taken at the expiration of three years from taking the degree of M.B. The fees for the degree of Doctor of Medicine, which entitles the possessor to the same elective privileges as the degree of Master of Arts, are £22.—*Ibid.*

